# Effect of the Pleiotropic Drug CNB-001 on Tissue Plasminogen Activator (tPA) Protease Activity in vitro: Support for Combination Therapy to Treat Acute Ischemic Stroke

**Published:** 2014-06-30

**Authors:** Paul A Lapchak, Paul D Boitano

**Affiliations:** 1 Departments of Neurology Advanced Health Sciences Pavilion, Los Angeles, USA; 2 Neurosurgery, Cedars-Sinai Medical Center, Advanced Health Sciences Pavilion, Los Angeles, USA

## Abstract

Current state-of-the-art acute ischemic stroke clinical trials are designed to study neuroprotectants when administered following thrombolysis; tissue plasminogen activator (tPA) is administered to patients within 3–4.5 hours of an ischemic event. Thus, in order to develop a novel neuroprotectant and move it forward to a clinical trial, it is important to assess the effects of the drug on tPA’s proteolytic activity in vitro, prior to extensive in vivo analysis.

In this study, we determined if CNB-001 [4-((1E)-2-(5-(4-hydroxy-3-methoxystyryl-)-1-phenyl-1H-pyrazoyl-3-yl)vinyl)-2-methoxy-phenol)], would affect, either enhance or inhibit tPA activity in vitro. In this tPA-inhibitor (plasminogen activator inhibitor-1; PAI-1 and 2,7-Bis-(4-Amidinobenzylidene)-Cycloheptan-1-One Dihydrochloride; tPA stop) controlled study, we used a chromogenic substrate (CH3SO2-D-hexahydrotyrosine-Gly-Arg-p-nitroanilide•AcOH) to study drug interactions in vitro, spectrophotometrically measuring protease released p-Nitroaniline from the substrate.

We found that PAI-1 (0.25 μM) and tPA stop (5 μM) significantly (p<0.0001) inhibited substrate release, by 98.6% and 83.4%, respectively, thus inhibiting tPA activity in vitro. In comparison, CNB-001 (0.7–7 μM) reduced tPA activity by 28–32%, with an extrapolated IC50 value of 65.2–704 μM. Thus, although high concentrations of CNB-001 does affects tPA activity in vitro, the study supports the use of CNB-001 in combination with tPA to treat stroke, However, CNB-001 should be administered following thrombolysis to promote neuroprotection and repair.

## Introduction

Acute ischemic stroke (AIS) is the fourth leading cause of death and the leading cause of adult disability in the United States with an estimated cost of $71 billion annually [1,2]. Despite this huge financial burden and impact to patients and to society, and allocation of billions of dollars of research and development funds to develop therapies, we still only have one single effective treatment strategy, the thrombolytic, tissue plasminogen activator (rt-PA) [3–5]. tPA is the only Food and Drug Administration (FDA) approved treatment for stroke [6–9] that improves clinical function measured using either the National Institutes of Health Stroke (NIHSS) scale or modified Rankin scores (mRS)[10–13].

We have developed a novel, potent, safe and effective drug candidate 4-((1E)-2-(5-(4-hydroxy-3-methoxystyryl-)-1-phenyl-1H-pyrazoyl-3-yl)vinyl)-2-methoxy-phenol), using phenotypic screening assays directed against some of the exacerbating mechanisms underlying initial cell death in stroke including mitochondrial dysfunction which reduces energy stores, and oxidative stress induced by intracellular glutathione depletion and glutamate-induced excitotoxicity [14,15]. While we have shown that CNB-001 supports cell survival activities measured using the in vitro assays described above [16], and is safe [17], it is also a potent 5-lipoxygenase inhibitor (5-LOX) [18], antiapoptotic and antioxidant [19], a negative regulator of inflammation (down-regulates, 5-LOX, cyclooxygenase-2 (COX-2), interleukin-6 (IL-6)) [20–22], and an activator of brain-derived neurotrophic factor (BDNF) and its signaling pathways [20]. The pleiotropic nature of the drug may provide maximal cellular protection and repair to the neurovascular unit [20,23–26] in vivo. Moreover, in vivo, we have found that CNB-001 promotes behavioral recovery when administered following embolic strokes in rabbits [20]. Since CNB-001 is an excellent candidate from a new class of compound, we are continuing to develop CNB-001 as a drug to be administered in combination with the only current FDA-approved treatment for stroke, tPA. In order to develop a treatment regimen for testing in an embolic stroke model [27], we first determined if CNB-001 would alter tPA activity in vitro using a sensitive assay.

## Materials and Methods

### Drug preparation

CNB-001 was synthesized by AQ BioPharma Co., Ltd. (Shanghai, China) according to Liu et al., [28]. CNB-001 was previously characterized as a neuroprotective molecule and neurotrophic factor with EC50 value of 0.7 μM [28] and a 5-LOX inhibitor with an IC50 value of 0.0765 μM (unpublished).

### Reagents

Tissue plasminogen activator chomogenic substrate (CH3SO2-D-HHT-Gly-Arg-pNA•AcOH; HHT=hexahydrotyrosine; pNA= p-nitroanilide) from purchased Sigma-Aldrich (Saint Louis, Missouri). Human recombinant tPA (Activase) was purchased from Genentech (San Francisco, California). 2,7-Bis-(4-amidinobenzylidene)-cycloheptan-1-one dihydrochloride (Pefabloc^®^ tPA/Xa; tPA-Stop) was purchased from Pentapharm Ltd. (Basel, Switzerland), and recombinant Human Plasminogen Activator Inhibitor −1 (PAI-1) was purchased from Sigma Inc. (St. Louis, MO).

### Methods

Enzyme assays were performed using a modification of the tissue plasminogen activator (tPA) chromogenic substrate product methods provided by Sigma-Aldrich Inc. (St. Louis, MO) so that the assay could be conducted in 96 well plates. The buffer system (Reagent 1) contained 30 mM tris-HCL, 30 mM imidazole and 130 mM NaCl. Reactions were measured using a SpectraMax M2 spectrophotometer maintained at 39°C (Molecular Devices, Sunnyvale, California). Change in absorbance per minute is reported herein (ΔA/min).

### Microplate tPA assay (96 well plate)

We adjusted the volumes, so that all reactions could be run in a 96 well plate with controls run in parallel to drug-treated groups. Briefly, 1 μl of tPA solution (equivalent to 580 IU or 1 μg, final concentration: 5 ng/mL) and 177 μl of Reagent 1 were preincubated at 39°C. 10 μl of chromogenic substrate solution (4 mM; final concentration 0.212 mM) was added to initiate the reaction, and ΔA/min was measured over 10 minutes at 1 minute intervals, or over 60 minutes at 5 minute intervals using a heated SpectraMax M2 spectrophotometer.

### PAI-1 inhibition assays

Plasminogen activator inhibitor 1(PAI-1) protein is the principal endogenous inhibitor of tPA [29–31]. We modified the chromogenic substrate procedure to be used in a 96 well plate. To analyze the effectiveness of the inhibitor, we used 2 μl of PAI-1 (final concentration: 0.247 μM), which was pre-incubated with 1μl of tPA and 175 μl of Reagent 1 at 39°C for 5 minutes. The reaction was then initiated using 10 μl chromogenic substrate solution (4mM stock concentration). ΔA/min was measured over 10 minutes at 1 minute intervals.

### tPA-STOP inhibition assays

Pefabloc^®^ tPA/Xa, or tPA-Stop, is a synthetic tPA inhibitor that has a Ki value of 0.035 μM for the fully active two-chain form of tPA (tc-tPA) [32], according to the product information data sheet provided by Pentapharm Inc. To analyze the effectiveness of the inhibitor, we used 5 μl of tPA-Stop (final concentration: 5 μM), which was pre-incubated with 5 μl of tPA and 175 μl of Reagent 1 at 39°C for 5 minutes. The reaction was then initiated using 10 μl chromogenic substrate solution (4mM stock concentration). ΔA/min was measured over 10 minutes at 1 minute intervals.

### CNB-001 interaction assays

Using the assays established using the procedures described above, we used 2 μl of CNB-001 (final concentration: 7 μM, 0.7 μM, 70 nM, 7 nM, and 0.7 nM), which was pre-incubated with 1 μl of tPA and 175 μl of Reagent 1 at 39°C for 5 minutes at 39°C The reaction was then initiated using 10 μl chromogenic substrate solution (4 mM stock concentration). ΔA/min was measured over 10 minutes at 1 minute intervals.

### Statistical Analysis

The studies were conducted in a manner with vehicle control, positive controls (inhibitors), randomized and blinded per current research study recommendations [33–37]. Statistical analysis using the unpaired t-test was conducted using GraphPad. Linear regression analysis was conducted using either Microsoft Excel or SIGMA Plot. IC50 values were extrapolated from SIGMA Plot graphs.

## Results

### tPA Chromogenic Substrate Assay

**Figure 1:**
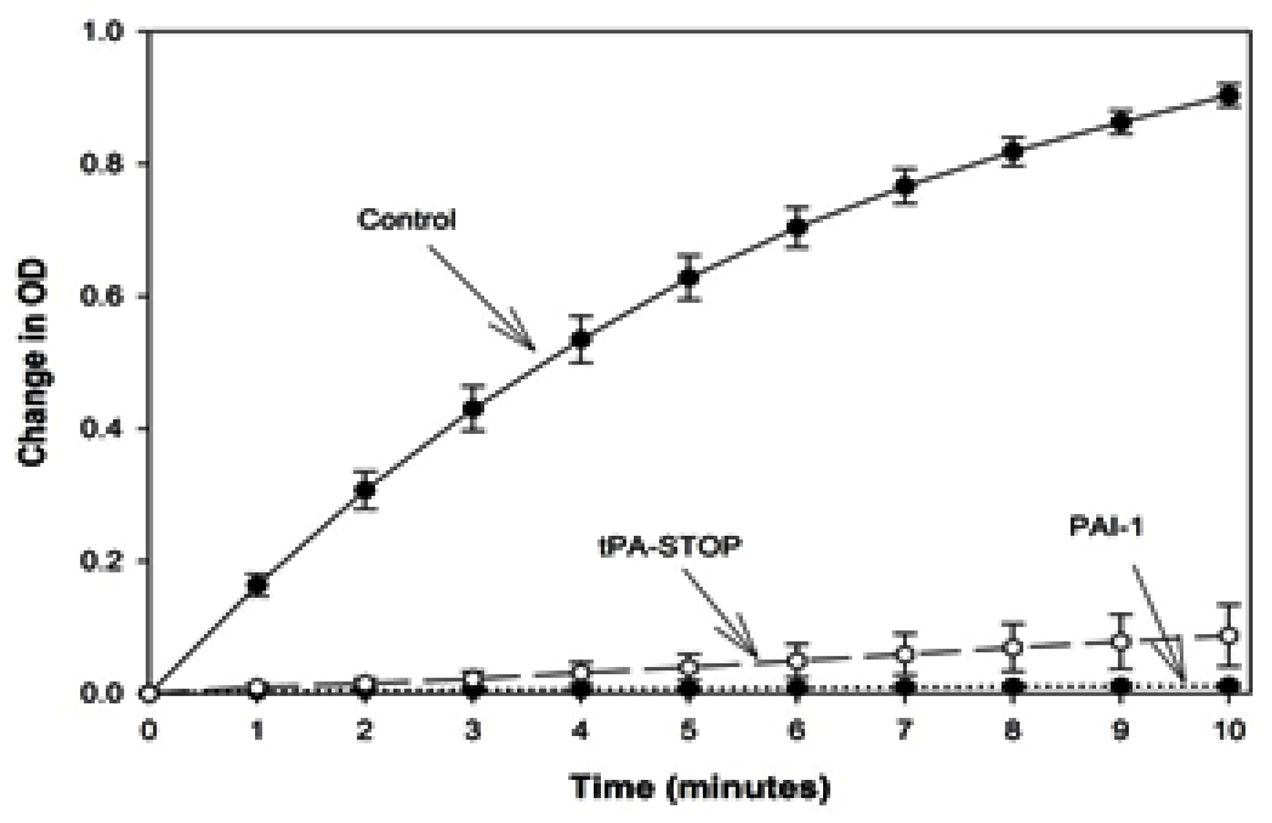
Figure 1A. tPA chromogenic assay time-course: tPA-STOP (5 μM-dashed line-open circle) and PAI-1 (0.25 μM- dotted line-closed circles) significantly inhibit tPA activity measured in vitro (Change in OD) compared to control (solid line- closed circles).

The first series of studies determined the baseline characteristics of the tPA activity assay, using a 10 minute assay. In Figure 1, linear regression analysis indicates an R2 value of 0.9634 for 0–5 minutes, with y=0.1046× and y=0.1335× and R2 value of 0.8476 for 0–10 minutes, respectively. Data is reported using both 0–5 and 0–10 minute assays. In the 60-minute assay, there was an increase in OD, i.e., cleavage of substrate out to 20 minutes after initiation of the assay; this then reached a plateau (not shown). Thus, the concentration of the chromogenic substrate used in the assay was not deemed to be a limiting factor. In subsequent experiments to study drug interactions, the assay duration was 10 minutes, and extracted 5 and 10 minute results are provided.

### Positive controlled study

**Figure 2:**
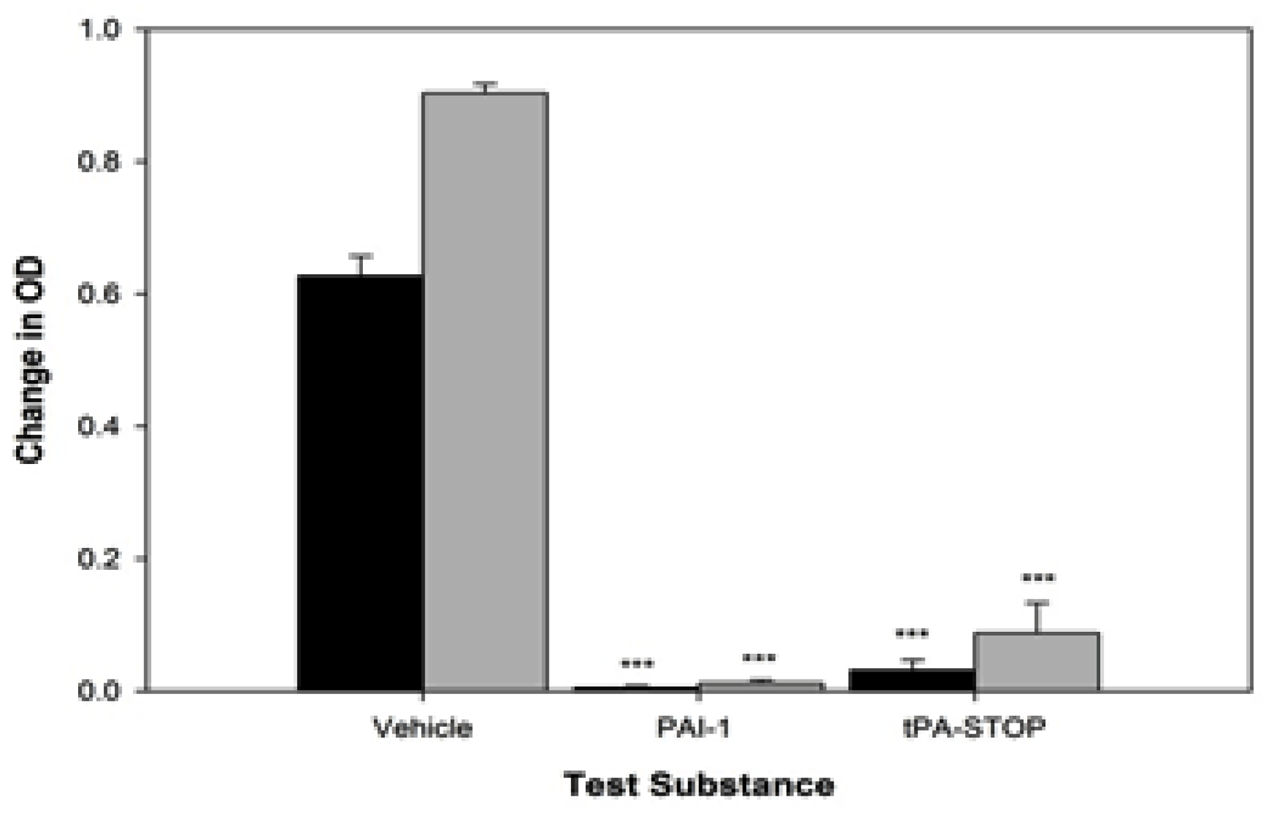
Quantitative tPA-STOP and PAI-1 inhibition analysis: Quantitation of the extent of tPA activity (Change in OD) inhibition by tPA-STOP (5 μM) and PAI-1 (0.25 μM). Both tPA-STOP and PAI-1 significantly (p<0.001) inhibit tPA activity by 83–98% compared to control (p<0.001) Black bars- 5 minute OD reading; Grey bars- 10 minute OD reading.

In Figure 1, we provide time-course data showing that the protease activity of tPA can be significantly inhibited under the specific conditions used in this assay by both PAI-1 (0.247 μM) and tPA-STOP (5 μM). As shown in Figures 1 and 2, PAI-1 produced 98% inhibition of tPA activity (p<0.001; t=14.0789), and tPA-STOP inhibited tPA by 83% (p<0.001; t=10.2323). There were no significant difference between PAI-I and tPA-STOP (p= 2.1501; t=2.1501).

### CNB-001-tPA Interactions

**Figure 3:**
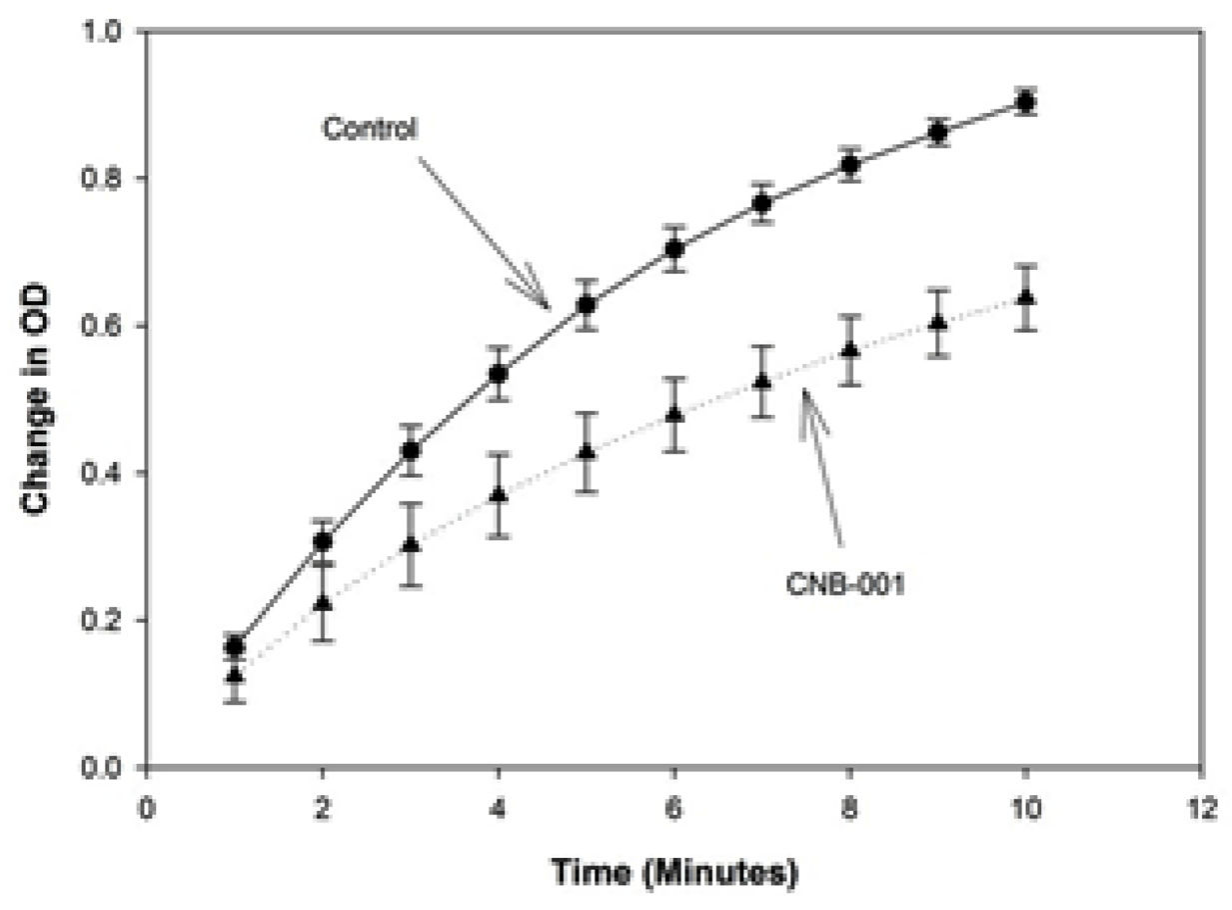
CNB-001 tPA interaction assay progression time-course: Effect of CNB-001 (7 μM- dotted line- closed triangle) on tPA activity measured in vitro compared to control (solid line- closed circle). CNB-001 produces modest inhibition of tPA activity that is most notable 5–10 minutes after initiation of the assay reaction.

In Figure 3, we provide time-course data curves for the effect of CNB-001 (7 μM) on tPA activity for the duration of the 10 minute assay. Under the condition used in this study, 7 μM CNB-001, which is 10X the EC50 for neuroprotection and neuroprotection using in vitro assays [16], reduced tPA activity by 26–32%.

**Figure 4:**
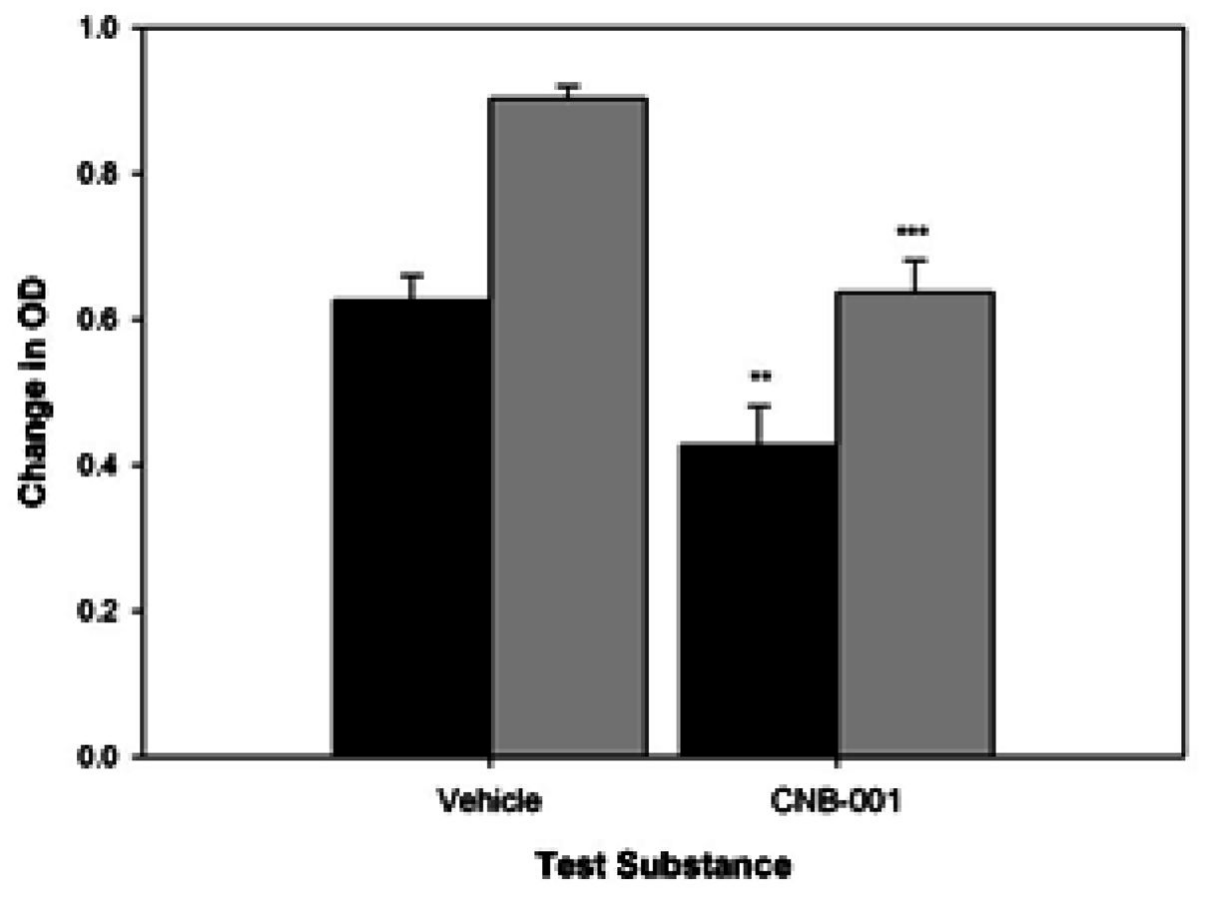
Quantitative tPA-STOP and PAI-1 inhibition analysis: Quantitation of the extent of tPA activity (Shown as Change in OD) inhibition by CNB-001 (7 μM). CNB-001 significantly (***p<0.001) inhibited tPA activity by 32% (**p<0.01, 5 minutes assay) and 26% (***p<0.001, 10 minutes assay) compared to control Black bars- 5 minute OD reading; Grey bars- 10 minute OD reading

**Figure 5:**
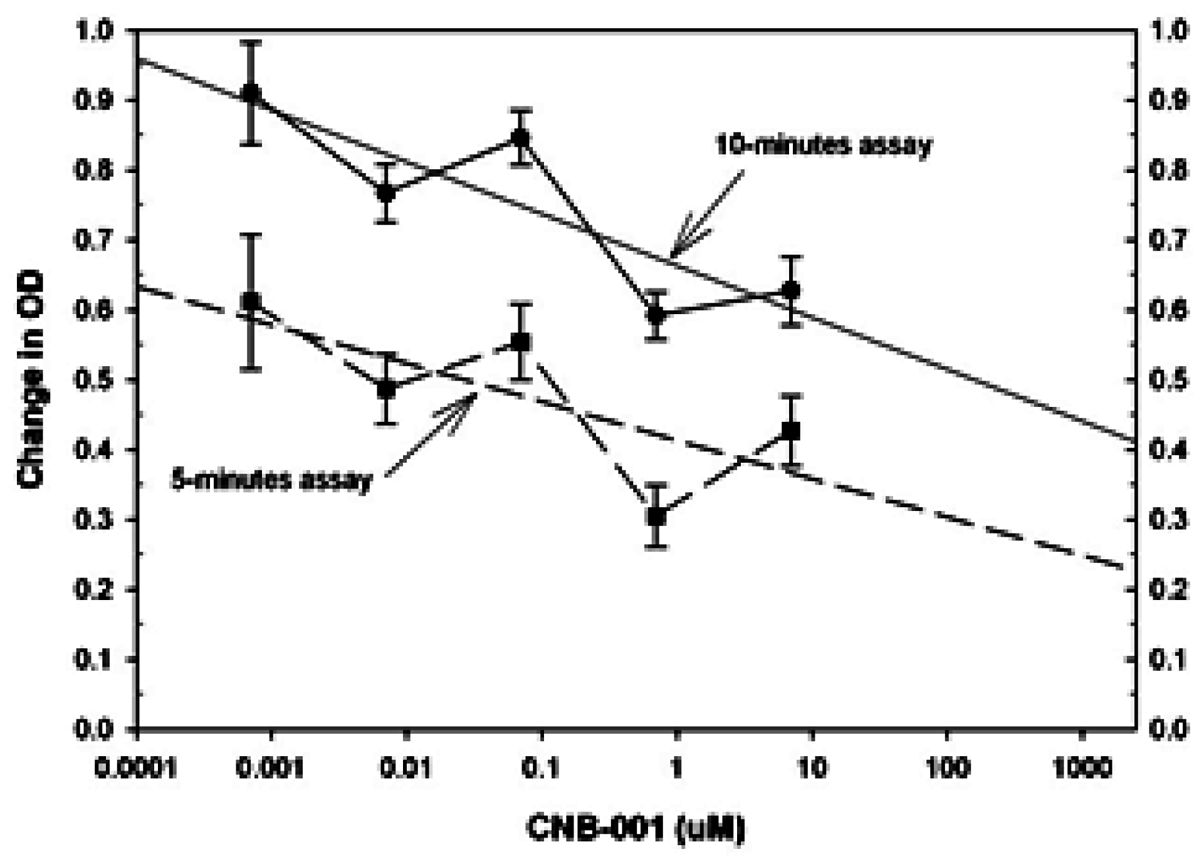
CNB-001 tPA interaction dose-response curves (IC50 extrapolation): Effect of CNB-001 (7 nM to 7 μM) on tPA activity measured in vitro compared to control. The results presented in the form of both 5 (dashed line- closed square) and 10 minutes (solid line- closed triangle) tPA assays. The diagonal lines are linear regression curve fits in order to extrapolate IC50 values. Control values for the assays are OD=0.62 and 0.90 for the 5 and 10 minute assays, respectively. Extrapolated IC50 values are 65.2 and 704 μM, respectively for the 5 and 10 minute assays

In Figure 4, we provide a dose-response curve for the effects of CNB-001 on tPA activity using CNB-001 concentrations as high as 7 μM. The baseline tPA activity data is for incubation of tPA in the presence of DMSO, which was used to solubilize CNB-001. We note that there was some effect of CNB-001 at the highest doses studied, with 26–32% inhibition of tPA activity. Linear regression analysis allowed us to use the CNB-001 dose-response to extrapolate inhibitory concentration for 50% effect (IC50) for both the 5 minute and 10 minute dose response curves (Figure 5). The IC50 values were 65.2 and 704 μM, respectively, both is excess (9.3 and 100.5 fold) of concentrations required for in vitro neuroprotective/neurotrophic activity.

## Conclusion

In this study, we determined the effects of a novel pleiotropic compound, CNB-001, on tPA activity in vitro, and compared the effects of CNB-001 to 2 commercially available tPA inhibitors, the small molecule tPA-STOP and the protein PAI-1. Both tPA-STOP and PAI-1 potently inhibited tPA activity by >83% using the chromogenic substrate assay that we have established. In contrast, CNB-001 at the highest concentrations tested produced a 26–32% reduction in tPA activity.

Current guidelines for effective translational drug development do not specifically recommend testing the effects of novel neuroprotectants on tPA activity in vitro [38–40]. Considering that tPA is the first and only choice therapeutic for stroke patients [3–5], and all newly developed neuroprotectants would eventually be used as a combination therapy (unless there are adverse interactions), the recommendation for extensive in vitro combination testing should be made and adhered to. As is clear from this study, a well-controlled in vitro study has provided important information that is clinically useful, because it offers guidance into the design of a combination treatment regimen trial. Additionally, if the treatment of stroke patients continues to evolve, then it is foreseeable that FAST track neuroprotection [41] and FAST track thrombolysis [42] would be useful to treat stroke patients in the field. This strategy could include the administration of tPA and an efficacious neuroprotectant of choice. Under either scenario, in vitro combination testing would be beneficial.

In conclusion, although CNB-001 does have some effect on tPA activity in vitro, the results of this study support the use of CNB-001 in combination with tPA to treat stroke. The only caveat is the timing for CNB-001 administration. If CNB-001 is to be used as a combination therapy in stroke patients, and it is imperative to have optimal tPA-induced recanalization, CNB-001 should be administered after tPA when thrombolysis is complete.
